# Endothelialization of Novel Magnesium-Rare Earth Alloys with Fluoride and Collagen Coating

**DOI:** 10.3390/ijms15045263

**Published:** 2014-03-25

**Authors:** Nan Zhao, Benjamin Workman, Donghui Zhu

**Affiliations:** Department of Chemical, Biological and Bio-Engineering, NSF Engineering Research Center-Revolutionizing Metallic Biomaterials, North Carolina Agricultural and Technical State University, Greensboro, NC 27411, USA; E-Mails: nzhao@aggies.ncat.edu (N.Z.); bdworkma@live.unc.edu (B.W.)

**Keywords:** magnesium, rare earth elements, corrosion, surface coating, biocompatibility

## Abstract

Magnesium (Mg) alloys are promising scaffolds for the next generation of cardiovascular stents because of their better biocompatibility and biodegradation compared to traditional metals. However, insufficient mechanical strength and high degradation rate are still the two main limitations for Mg materials. Hydrofluoric acid (HF) treatment and collagen coating were used in this research to improve the endothelialization of two rare earth-based Mg alloys. Results demonstrated that a nanoporous film structure of fluoride with thickness of ~20 μm was formed on the Mg material surface, which improved the corrosion resistance. Primary human coronary artery endothelial cells (HCAECs) had much better attachment, spreading, growth and proliferation (the process of endothelialization) on HF-treated Mg materials compared to bare- or collagen-coated ones.

## 1. Introduction

Magnesium (Mg)-based alloys are promising biomaterials for biomedicine and tissue regeneration [1–4]. Some of the key advantages of the Mg-based alloy include light weight, good biocompatibility and biodegradability [5,6]. However, the two main drawbacks of Mg materials are high corrosion rate and insufficient mechanical strength [7,8]. A variety of techniques have been explored to improve their mechanical properties, corrosion resistance and biocompatibility, namely alloying, protective coating and surface treatment [9]. In design of degradable biomaterials, elements with no or minimal potential toxicity, as well as those that can improve mechanical and corrosion properties should be used. Therefore, the main alloying elements as documented are Ca, Mn, Zn, Sn, Si, Al, Bi, Li, Sr, Zr, Sb, Y, and rare earth (RE) elements [10,11].

For cardiovascular applications, Mg-based biodegradable stents emerged recently and represent the most advanced stent scaffolds [12–16]. For example, an absorbable metal stent (AMS, Biotronik) currently under clinical investigation is reportedly made of an alloy containing Mg, Zr, Y and RE, although the exact composition of the alloy has not yet been disclosed [16]. Another clinical trial was reported on Mg and RE-based DREAMS cardiovascular stents from Biotronik with promising outcomes [14]. Nonetheless, most of the current cardiovascular stents exhibit suboptimal biocompatibility and sequential thrombosis formation due to incomplete stent endothelialization [17]. Late or very late thrombosis with adverse clinical outcomes may occur as a result of delayed or absent stent endothelialization.

Previous studies from us as well as many others demonstrated Mg-RE alloys possessed remarkably better mechanical strength and corrosion resistance compared to pure Mg [11]. In this study, we extended our research to explore the endothelialization on Mg-RE stent biomaterials through surface modification, *i.e.*, fluoride conversion and collagen coating. Fluoride conversion coating has attracted much attention due to its potential to improve the corrosion resistance of metallic alloys [18–22]. Fluoride is essential for human normal dental development [23], and studies in dogs have shown that in the early phase of healing, fluoride-modified surfaces can promote osteointegration following implantation [24]. Fluoride coating can improve the corrosion resistance of Mg or its alloys by forming a thin layer of MgF_2_ [9]. Chen *et al.* reported that the corrosion resistance of pure Mg after hydrofluoric acid treatment was improved 30 to 40 times better than uncoated Mg [25]. In another study conducted by Seitz *et al.*, L929 and MSC cells showed better viability and proliferation on the MgF_2_ surface [26]. In this study, we also used natural collagen coating as a comparative control because collagen, the main composition of extracellular matrix (ECM) protein, is effective in accelerating cellular adhesion, spreading, and promoting cell proliferation. Despite numerous studies of collagen coatings and HF modification on different biomaterials, including titanium, steels, and ceramics [27–29], there is still no report on their effects of endothelialization on Mg-based alloys. The goal of this study was to evaluate the fluoride coated material and to compare the results to collagen coated and uncoated Mg-RE alloys for endothelialization, a healing process after stent implantation and the ultimate solution for in-stent restenosis.

## 2. Results and Discussion

Two novel rare earth-based Mg alloys, R1 (MgYZrRE) and R2 (MgZnYZrRE), gifts from Drs. J. Sankar and Z. Xu were used in this study with pure Mg as a control.

### 2.1. Collagen Assembly on Alloy Surface

**Figure 1.**
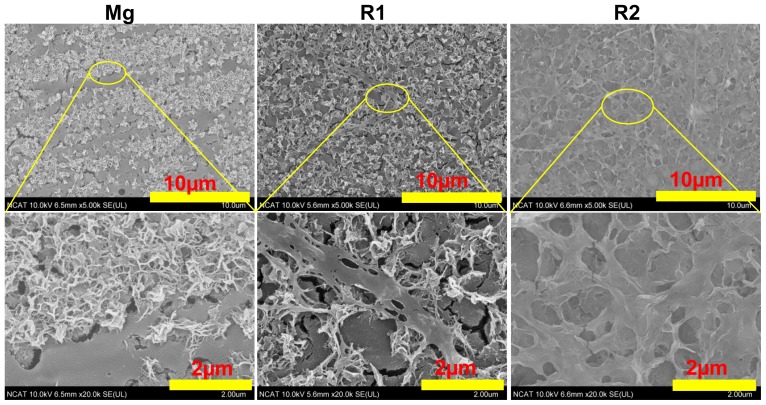
SEM images of type I collagen coating morphologies. All three materials were incubated with 50 μL 200 μg/mL collagen solutions in DPBS for 2 h.

Figure 1 shows the surface morphology of the three materials after coated with collagen. Collagen on pure Mg self-assembled into small fibrils, with lengths less than 2 μm. Most of the small fibrils mingled with others, forming some network structures. On the R1 surface, some long fibers more than 10 μm in length were observed beside the small fibrils. A few of the fibrils intertwined with others forming thicker bundles. In contrast, a collagen sheet mixed with the long collagen fiber covered the entire surface of R2. The different morphology structure may be caused by the different degradation rate of the three materials. The degradation rates for pure Mg, R1 and R2 in static simulated body fluid were measured to be 6.02, 2.26, and 1.50 mm/year, respectively (Table 1). Slower degradation rates could reduce the local concentration of degradation products including Mg and rare earth ions. It was shown that electrolytes such as MgCl_2_, NaCl, and KCl could affect the process of collagen self-assembly [30]. But it is unclear how rare earth elements affect the process of collagen self-assembly. It appeared that collagen exhibited better assembly structure on the RE-based alloys, indicating that either the degradation rate in Mg-RE alloys was too slow to affect collagen assembly or RE presence had little impact on collagen self-assembly. The pH value is another factor that might contribute to the different assembly results. The pH value of the assembly environment could determine the electrostatic charge in the collagen monomers. It was believed that at isoelectric point (pI) of pH 9.3, collagen could exhibit optimal assembly results [31]. However, absorption of ions on collagen molecules could lead to the shift of the pI to a lower pH value, therefore, the optimal condition for collagen assembly is often at pH of 7–8 [31]. Higher pH and metal ion absorption, resulting from fast degradation in pure Mg, could be the reason why only small collagen fibrils were present. Repulsive forces between collagen molecules on the Mg surface inhibited the assembly of small fibrils into long ones. The microstructures of collagen on R1 and R2 were also different.

**Figure 2.**
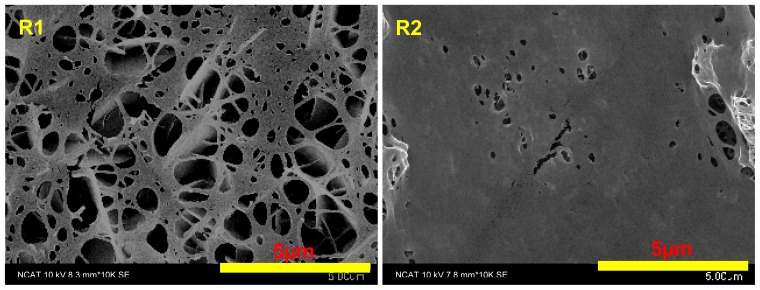
SEM images of collagen on pure Mg. Collagen stock solution was diluted by R1 (**Left**) and R2 (**Right**) extract solution and then coated on the pure Mg surface.

Next, collagen stock solution diluted by R1 and R2 extract solutions was coated on the pure Mg. The morphology of collagen (Figure 2) diluted by R1 and R2 extract solution was much different from that of collagen in DPBS (Figure 1, left panel), indicating that the electrolytes released from the degradation process could affect the assembly of collagen. A layer of collagen sheet with some small cavities was observed in both. R1 has larger and more cavities compared with R2. The formation of hydrogen bubbles during the corrosion process may have caused such a difference, as they prevented collagen absorption onto the surface of Mg materials. The presence of Zn in R2 could reduce hydrogen gas production because zinc ions in solution can compete with Mg ions to form Zn(OH)_2_, thereby reducing the production of hydrogen gas [1].

The typical native collagen fibril structure, D-period of 67 nm [32], was absent from all the groups. D-period which plays very important role in biological functions and mechanical functions was formed by quasi-hexagonal packing of collagen monomer [27,33]. This absence of D-period could be due to the lack of potassium ion necessary for native D-period structure [30]. Future research is needed to clarify the exact mechanism of collagen self-assembly on Mg alloy surfaces.

### 2.2. Fluoride Formation on Alloy Surface

**Figure 3.**
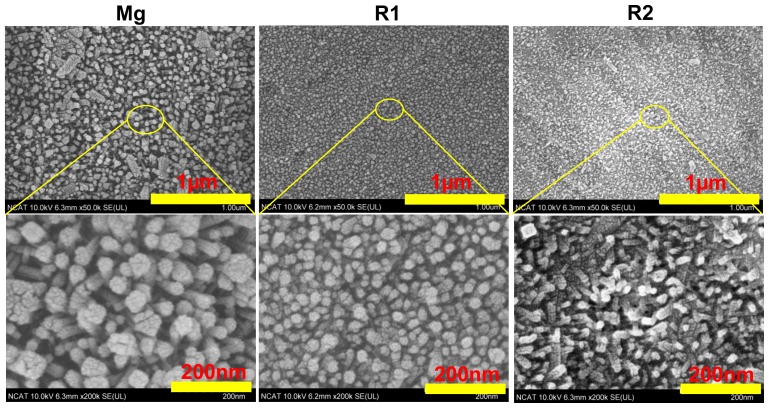
SEM images of fluoride coating morphologies. All the materials were treated with HF solution for 3 days.

Surface morphology of the three materials after treatment with HF for 3 days was shown in Figure 3. Pure Mg surface was converted into a layer of granular and columnar structure. The diameter of the granules was ~50 nm and the length of the columnar structure was ~200 nm. In both R1 and R2, the granular structures from the converted layers were much smaller than that on the pure Mg surface. Compared to pure Mg, grain size refinement by rare earth elements or direct reactions between rare earth elements with HF may cause such finer porous structures on HF-treated Mg-RE alloys. The addition of Zn in R2 could be the reason for the different alignment of the granular structure. Similarly, Mao *et al.* showed that Mg-Nd-Zn-Zr alloy, after treatment with HF solution for 12 h, forms a porous layer of MgF_2_ on its surface [21]. Also, it was shown that the bonding strength of the interlayer of MgF_2_ in pure Mg treated with HF was found to be 34 MPa [34]. Wan *et al.* also reported a super-hydrophobic porous surface created by 1% HF treatment [35]. It is believed that the porous structure played an important role in trapping air, which leads to the hydrophobic surface. The porous structure with smaller cavities was observed in Figure 3. MgF_2_ is insoluble in water and the small cavities among the granular structure were able to trap air. The capability of the HF modified layer to ameliorate Mg degradation is mainly dependent on the size of those granular structures. The smaller cavities on the MgF_2_ layer, the more efficient they are at trapping air. Another notion, according to a previous study [26], is that the MgF_2_ layer on the material surface might be brittle, thus a modification of the mechanical properties is to be expected. Therefore, one needs to be cautious when applying this HF treatment for balloon expandable stent materials.

**Figure 4.**
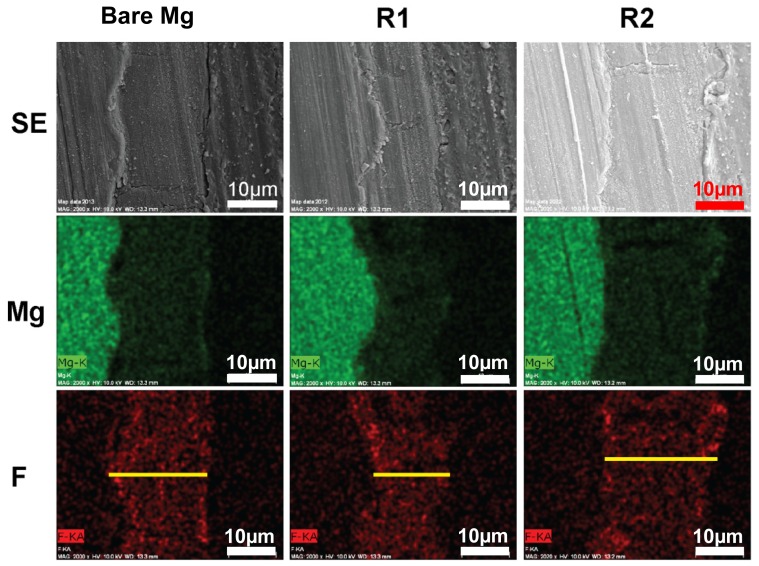
EDS Mapping for cross section of fluoride coating. First row: SEM photos displaying cross sections of magnesium fluoride coating in epoxide resin; Second row: magnesium (Mg, green); Third row: fluorine (F, red). Yellow lines indicate the thickness of fluoride coating (Scale bar: 10 μm).

The coating thickness in all samples was about 20 μm (Figure 4), suggesting that alloying with RE elements will not affect the thickness of the MgF_2_ layer. Transection images of SEM showed that there was a distinctive boundary between Mg substrate and the MgF_2_ modified layer, most likely caused by chemical composition change and mechanical mismatch between the surface layer and the Mg substrate.

### 2.3. Corrosion and pH Change

**Figure 5.**
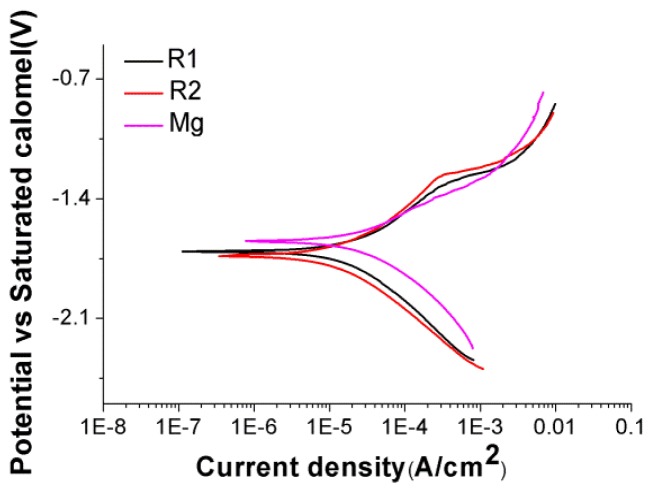
DC Polarization curves of three materials in HBS.

Electrochemical corrosion tests were carried out in Hank’s balanced solution (HBS) at room temperature. DC polarization curves of three bare materials were shown in Figure 5 and corrosion rates for all samples were summarized in Table 1. R2 had the slowest corrosion rate while pure Mg control had the fastest corrosion rate. Pure Mg alloying with rare earth elements reduced the corrosion rate in HBS. The pH value of culture media soaked with all the materials for 3 days was measured. For bare pure Mg the pH rose to 8.70, while HF treated R2 had the lowest pH value of 7.56 after 3 days. Among bare material, HF treated group and collagen treated group, HF treated material showed the least amount of pH change. This result was in good agreement with many previous studies, as supporting HF treatment was an effective way to increase corrosion resistance of Mg-based alloy [19,22,35]. Collagen coating had less effect on preventing Mg degradation so that pH in the collagen coated group was still higher than 8 after 3 days, which is beyond the maximum pH that endothelial cells can tolerate. Also, high pH could lead to fast degradation of collagen fibers, which in turn could interact with magnesium degradation.

### 2.4. Direct Endothelialization on Alloy Surface

**Figure 6.**
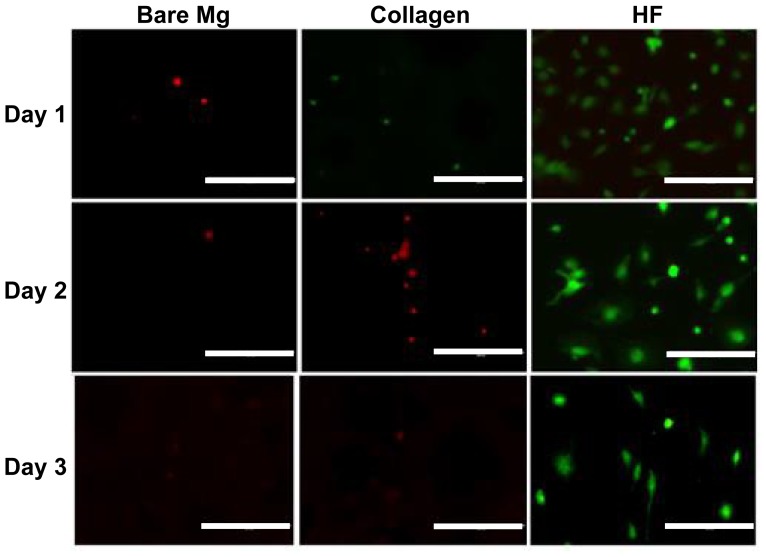
Endothelial cells cultured on bare Mg, collagen coated Mg, and HF treated Mg from day 1 to day 3 (Scale bar: 10 μm).

The endothelial layer is the most inner layer of the blood vessel and separates blood plasma from coagulation initiation molecules within the medial layer of the blood vessel [36]. For stent material, endothelial cell coverage is an important indicator, which to a large extent could represent the performance of a stent material [37,38]. The Live/Dead kit including calcein AM and Ethidium homodimer-1 (EthD-1) was used to test how cells directly interact with alloys and coatings. Calcein AM could be metabolized by ubiquitous intracellular esterase activities resulting in the presence of green fluorescence in live cells. EthD-1 is excluded by the intact plasma membrane of live cells. Upon binding to nucleic acids, the emission intensity of EthD-1 at 635 nm undergoes a 40-fold enhancement. Representative images of direct endothelialization for 1, 2, and 3 days on bare Mg, Mg coated with collagen and Mg treated with HF are shown in Figure 6.

HF treated Mg yielded the most attached and viable cells (green) in all groups. Cell density slightly decreased from the 1st to 3rd day on pure Mg treated with HF. For the non-treated pure Mg control group, some dead cells (red) were still observed on the very first day but none were observed either on day 2 or 3. A few live cells appeared on the surface of collagen coated Mg but the density was much lower than that on Mg treated with HF. In addition, on days 2 and 3 only, dead cells could be seen on the collagen coated group. Collagen coating was used as positive control in this study since it is the most abundant extracellular matrix protein that provides adhesion points for cell attachment and migration. Ao *et al.* showed that type I collagen covalently combined with titanium enhanced cell-material interactions and improved hMSC attachment, proliferation, and differentiation [39]. In addition, collagen-coated Ti could promote expression of osteoblast phenotype and enhance bone formation around the implants [40]. However, on the Mg surface, collagen coating did not show much improvement for HCAECs attachment and proliferation. This is probably due to the different corrosion rate and corrosion mechanism. As the degradation of Mg alloys progressed, increased pH and excess alloying element ions could affect the 3-D structure of biomolecules, leading to the failure of recognition between extracellular matrix proteins and the cell membrane receptors. In addition, hydrogen gas production during the corrosion process could form air bubbles on the Mg surface, which may have prevented both biomacromolecule attachment and cell adhesion.

**Figure 7.**
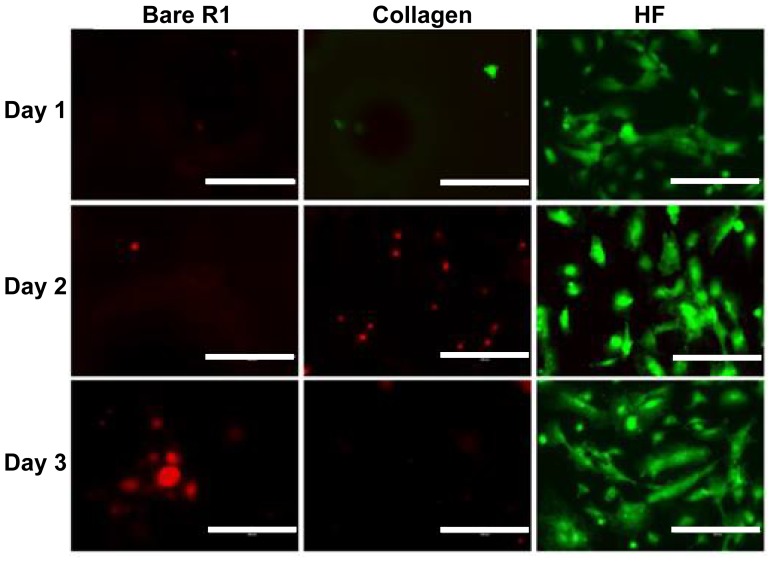
Endothelial cells cultured on bare R1, collagen coated R1 and HF treated R1 alloys from day 1 to day 3 (Scale bar: 10 μm).

Figure 7 shows the representative images of direct endothelialization on bare R1, R1 coated with collagen and R1 treated with HF. Results were similar to that of pure Mg group. Fluoride treated R1 had the most viable cells while bare R1 had the least. Better cell attachment was shown in the HF treated R1 group on the 1st day compared to the same one in the pure Mg group (Figure 6). Also, cell density on the 3rd day in HF treated R1 group did not decrease, unlike the case with pure Mg, which might exclude the possibility that decreased cell density on HF treated Mg was caused by fluoride. In addition, the finer porous structures on R1 that could trap air for a longer period of time may protect the degradation of Mg substrate for a longer time period.

**Figure 8.**
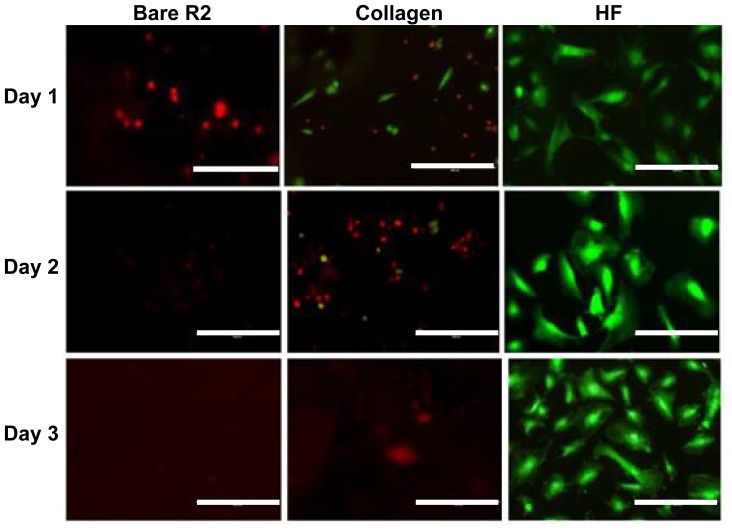
Endothelial cells cultured on bare R2, collagen coated R2 and HF treated R2 alloys from day 1 to day 3. (Scale bar: 10 μm).

Cell attachment and proliferation on the R2 group (Figure 8) were very similar to that in the R1 group (Figure 7). The most different one was the collagen-coated group, and much better endothelialization was observed compared with R1 and the pure Mg groups. Some fully spreading cells appeared at the very first day, which demonstrated that collagen could improve cytocompatibility to a certain degree. The preferable cell attachment on collagen coated R2 was most likely due to the denser layer of the collagen sheet separating cells from contacting with the alloy surface, which was absent from pure Mg and R1 (Figure 1). On the second day, most cells were dead and only a few cells were still alive in a stressed condition, and this could be caused by alloy degradation, hydrogen air bubble formation and higher pH. In addition, higher viable cell density was observed on day 3 than on days 1 and 2 for the fluoride coated R2, suggesting cell growth and proliferation which were absent for all other groups.

## 3. Experimental Section

### 3.1. Alloy Preparation

Pure Mg (99.97%) and Mg based alloys of MgYZrRE, MgZnYZrRE (RE includes Gd and Dy, denoted as R1 and R2) were gifts from Drs. J. Sankar and Z. Xu at NSF Engineering Research Center-Revolutionizing Metallic Biomaterials. Mg materials were cut (Techcut 5, Allied High Tech Products, Rancho Dominguez, CA, USA) into 10 × 10 × 1 mm pieces. Subsequently, the materials were polished (EcoMet 250 Grinder, Buehler, Lake Bluff, IL, USA) on each side using 180, 400, 600, 800, 1000, and finally 1200 grit abrasive papers with isopropyl alcohol (Sigma-Aldrich, St. Louis, MO, USA). The samples were then immersed in acetone (Sigma-Aldrich, St. Louis, MO, USA) and cleaned for five minutes in an ultrasonic bath (M2510 Ultrasonic cleaner, Branson Ultrasonics, Danbury, CT, USA). Following acetone cleaning, the samples were immersed in 100% ethanol (Sigma-Aldrich, St. Louis, MO, USA) for 5 min and allowed to air dry for 15 min. Each side of the alloys was sterilized by UV for 30 min. For all the experiments, at least three replicates were used (*n* ≥ 3).

DC polarization was carried out by a three electrode cell manufactured by Gamry instruments (Gamry Ref 600, Gamry Instruments, Warminster, PA, USA). The reference electrode, counter electrode and working electrode were saturated calomel, platinum and testing alloy. After immersion for a short time in Hank’s Balanced Solution (HBS, Invitrogen, Grand Island, NY, USA), DC polarization tests were conducted at a rate of 5 mV/s from −0.50 to 1.0 V with respect to the open circuit potential. All the DC polarization data were fitted and analyzed by Echem Analyst 6.0 (Gamry instrument, Warnubster, PA, USA). The corrosion rate was calculated as described previously [41].

### 3.2. Fluoride Coating

The prepared Mg samples were immersed horizontally in 1.0 mL of 47%–51% hydrofluoric acid (Sigma-Aldrich, St. Louis, MO, USA) for 3 days. At day 3, the alloys were removed from hydrofluoric acid (HF) and allowed to air dry. The SU8000 SEM (Hitachi, Schaumburg, IL, USA) was used to measure the surface morphologies of the alloys, and the Quantax EDS for SEM (Bruker, Ewing, NJ, USA) was used to measure the alloys’ thicknesses.

### 3.3. Collagen Coating

Rat tail type I collagen (3 mg/mL) was obtained from life technologies, USA. The stock collagen solution was diluted with DPBS (Invitrogen, Grand Island, NY, USA) to the final concentration of 200 μg/mL, and then placed onto the prepared material surfaces. Following surface coating, the alloys were incubated in a humidified incubator (Heracell 150 I, Thermo, Marietta, OH, USA) at 37.0 °C with 5.0% CO_2_ for two hours. After two hours, samples were washed with DPBS 3 times to remove unattached collagen followed by gradient ethanol dehydration. R1 and R2 extract solution were prepared by soaking alloys in DPBS with a ratio of 1.25 mL/cm^2^ in a humidified atmosphere with 5% CO_2_ at 37 °C for 2 h. Then collagen stock solution was diluted by the extract solutions to the final concentration of 200 μg/mL and then coated on pure Mg surface. Samples were dehydrated as mentioned before. Coating morphology was observed by SEM.

### 3.4. pH Assessment

Pure Mg (99.97%), R1, R2 alloys and corresponding collagen and HF treated materials were soaked in serum free endothelial culture medium (ECM, ScienCell, Carlsbad, CA, USA) for 3 days, and then the pH of each solution was measured by pH meter (Eutech, Vernon Hills, IL, USA).

### 3.5. Cell Culture

Human Coronary Artery Endothelial Cells (HCAEC) was purchased from ScienCell Research Laboratories (Carlsbad, CA, USA). HCAECs were expanded in complete endothelial culture medium (ScienCell, Carlsbad, CA, USA) with 10% fetal bovine serum, 100 U/mL penicillin and 100 μg/mL streptomycin on fibronectin coated 75 mL flasks (BD Biosciences, San Jose, CA, USA) at 37 °C in humidified incubator with 5% CO_2_. A fourth passage was used for this experiment. Culture medium was changed 24 h after cells were transferred into a new flask and then changed every 2 days.

### 3.6. In Vitro Endothelialization

A total of 50 μL endothelial cell (50,000 cells/mL) with serum free culture medium was pipetted onto the surface of each sample in a 24-well tissue culture plate (BD Biosciences, San Jose, CA, USA). Thereafter, the cells were allowed to attach for fifteen minutes. Then all the materials were gently washed with DPBS 3 times to remove the unattached cells. Thereafter, each alloy was immersed in 2.0 mL serum free culture medium, and the HCAECs were incubated in a humidified environment at 37.0 °C, 5.0% CO_2_. LIVE/DEAD Viability Kit (Invitrogen, Grand Island, NY, USA) was used to test cell attachment and viability at 1, 2, and 3 days, respectively. Culture medium was changed every 24 h. Culture medium was removed from the wells, and the materials were incubated for 30 min with 1.0 mL DPBS containing 20 μM Ethidium Homodimer-1 and 5 μM Calcein AM. Following incubation, cells were imaged under digital inverted light microscope (EVOS, Advanced Microscopy, Bothell, WA, USA).

## 4. Conclusions

Collagen and HF coatings were successfully prepared on rare earth-based Mg alloys intended for cardiovascular applications. HF treatment could modify the Mg surface into a nanoporous layer. The size and structure of the modified layer are dependent on the chemical composition of the alloy. In comparison to the non-coated Mg samples, both coatings significantly decreased the degradation rate with HF having the lowest rate of degradation. As a result, HF treated material demonstrated better endothelial cell attachment and proliferation than bare Mg or the same material coated with collagen. Such better endothelialization on HF coated materials could result from a combined effect including slower degradation, less pH change, less hydrogen gas release and nanoporous structures on the surface for easier cell attachment. These findings indicate that the HF coating prepared in the current study is promising for controlling the biodegradation and improving the cytocompatibility of rare earth-based Mg alloys. However, further cell and animal studies are necessary to translate such Mg implants to clinical applications.

## Figures and Tables

**Table 1.** Corrosion rate and pH of culture media after incubation with different materials for 3 days.

Metal	Treatment	pH	Corrosion rate (mm/year)
Mg	Bare material	8.70 ± 0.14	6.02
	Collagen	8.51 ± 0.05	
	HF	8.10 ± 0.03	
R1	Bare material	8.40 ± 0.13	2.26
	Collagen	8.26 ± 0.06	
	HF	7.76 ± 0.07	
R2	Bare material	8.35 ± 0.11	1.50
	Collagen	8.36 ± 0.08	
	HF	7.56 ± 0.07	
